# Clinical Phenotypes in Relation to Outcomes in Heart Failure Patients With Cardiac Resynchronization Therapy and Defibrillators (CRT‐D): An Unsupervised Cluster Analysis

**DOI:** 10.1111/jce.16727

**Published:** 2025-05-19

**Authors:** Luigi Gerra, Tommaso Bucci, Arnaud Bisson, Alexandre Bodin, Bertrand Pierre, Giuseppe Boriani, Gregory Y. H. Lip, Laurent Fauchier

**Affiliations:** ^1^ Liverpool Centre for Cardiovascular Science at University of Liverpool Liverpool John Moores University, and Liverpool Heart and Chest Hospital Liverpool UK; ^2^ Cardiology Division, Department of Biomedical, Metabolic and Neural Sciences University of Modena and Reggio Emilia, Policlinico di Modena Modena Italy; ^3^ Department of General and Specialized Surgery Sapienza University of Rome Rome Italy; ^4^ Cardiologie Centre Hospitalier Universitaire Trousseau Tours France; ^5^ Danish Center for Health Services Research, Department of Clinical Medicine Aalborg University Aalborg Denmark; ^6^ François Rabelais University Tours France

**Keywords:** cardiac resynchronization therapy, cluster analysis, heart failure, mortality, phenogroups

## Abstract

**Background:**

Patients with heart failure undergoing cardiac resynchronization therapy (CRT) are a heterogenous and complex population.

**Objective:**

To identify different clusters of patients with CRT‐D and to evaluate the associations with clinical outcomes, using cluster analysis (CAs).

**Methods:**

Three agglomerative hierarchical CAs were performed in CRT‐D patients seen between 2010 and 2019 in French hospitals. Associations between clusters and death at 1 year and death during the whole follow‐up (FU) were evaluated.

**Results:**

The study included 23 029 CRT‐D patients, who were analyzed in three ways, as follows: the first group was a 50% random sample of all patients (*n* = 11 514), the second group included patients who were dead at 1 year (*n* = 1604) and the third group included those alive at 3 years FU (*n* = 14 228). A CA was performed on each group of patients.

Four clusters were identified: *Cluster 1* corresponded to the low‐risk phenotype; *Cluster 2* to patients with coronary artery disease (CAD) with few cardiovascular (CV) risk factors and comorbidities; *Cluster 3* included patients with CV risk factors and comorbidities, but low CAD; *Cluster 4* corresponded to clinically complex phenotype (CAD with CV risk factors and comorbidities). Compared with Cluster 1, Clusters 2, 3, and 4 were independently associated with an increased risk of all‐cause death at 1‐year FU and during the whole FU (Cluster 2: hazard ratio (HR) 1.21, 95% confidence interval (CI) 1.08–1.36; Cluster 3: HR 1.15, 95% CI 1.04–1.26; and Cluster 4: HR 1.79, 95% CI 1.65–1.96).

**Conclusion:**

CAs identified four statistically driven groups of CRT recipients, with specific clinical phenotypes and associated with different risks for all‐cause death.

## Graphical abstract

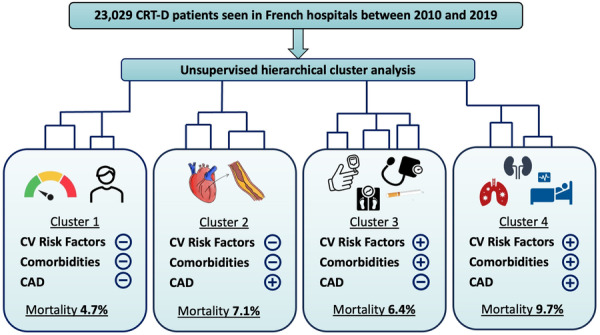

## 1 Introduction

Since its introduction in 2000s, cardiac resynchronization therapy and defibrillator (CRT‐D) has become one of the most effective therapeutic options for heart failure (HF) patients with reduced ejection fraction (HFrEF) and specific clinical features (i.e., left ventricular ejection fraction (LVEF) ≤ 35–40%, wide QRS complex, and symptoms, despite optimal medical therapy (OMT)) [1]. In these patients, CRT‐D has been associated with functional status improvement [2], echocardiographic remodeling [3], and reduction of all‐cause mortality or hospitalizations [4, 5, 6, 7]. Therefore, CRT‐D is strongly recommended in international guidelines on HF management [8, 9]. However, the evidence suggests that about one‐third of HF patients with HFrEF do not show a favorable response to CRT‐D, highlighting the need for a better selection of patients who can benefit from this treatment.

Unsupervised cluster analysis identifies complex categories without investigator supervision by dividing patients into homogenous groups based on similar clinical features [10]. This data‐driven method allows to reveal different clinical phenotypes in a population that has been previously considered homogenous. Therefore, cluster analysis might help to shed light on the complexity of CRT‐D candidates, by identifying clinical phenotypes that may impact clinical outcomes and responsiveness to CRT‐D implantation.

To date, few cluster analyses have been conducted in these patients [11, 12]. In this large French national registry, we sought to investigate the risk of all‐cause death in CRT‐D recipients based on the presence of different clinical phenotypes identified by cluster analyses.

## 2 Methods

### 2.1 Study Population

**Figure 1:**
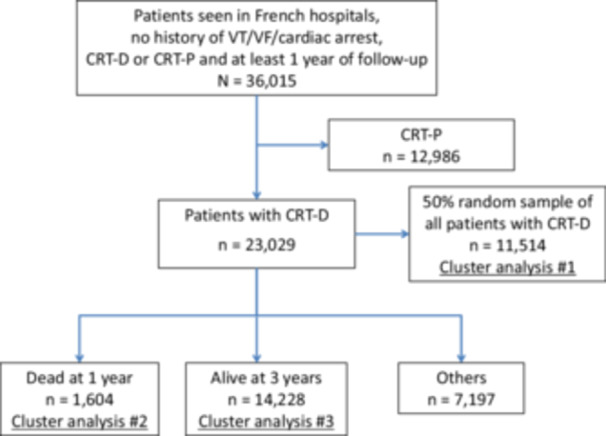
Flowchart of the study patients.

The study initially included HF patients with CRT‐D or CRT‐P seen in French hospitals between 2010 and 2019 with no history of ventricular tachycardia (VT), ventricular fibrillation (VF), or cardiac arrest. Patients with CRT‐P were subsequently excluded from the analysis, and the remaining patients were divided into three groups. The first group (group 1) was a 50% random sample of all patients with CRT‐D, group 2 included patients who died within 1 year of follow‐up and the third group (group 3) included those CRT recipients alive at 3 years (Figure 1). We defined “cardiovascular risk factors” as traditional modifiable conditions associated with atherosclerosis (arterial hypertension, diabetes, dyslipidemia, smoking), while “comorbidities” included diseases such as chronic kidney disease (CKD), peripheral arterial disease (PAD), chronic obstructive pulmonary disease (COPD), thyroid diseases, and previous cancer.

### 2.2 Follow‐Up and Outcomes

During the study period, the in‐hospital endpoints were obtained from medical records in the hospitals during follow‐up by analyzing the PMSI codes for each patient. They were evaluated with follow‐up starting from the first hospitalization until the date of each specific outcome or date of last news in the absence of the outcome. The primary endpoint was all‐cause death at 1‐year follow‐up and all‐cause death during the whole study period.

### 2.3 Cluster Analysis

Three separate cluster analyses were performed in the three groups of patients (group 1–2–3). For each variable a missing data rate above 25% was the cut‐off for exclusion, while variables with a missing data rate ≤ 25% were imputed using the “Multivariate Imputation by Chained Equations” algorithm (S. van Buuren, K. Groothuis‐Oudshoorn, *Mice: Multivariate Imputation by Chained Equations in R*, Journal of Statistical Software 45 (2011): 1–67.). Thirty‐five baseline clinical variables were therefore used for the analysis (Table 1).

**Table 1:** Baseline characteristics of patients with CRT‐D seen in French hospitals (2010–2019) with no history of previous VF/sustained VT/cardiac arrest and at least 1 year of follow‐up.

	Total	Death at 1‐year FU	Alive at 3‐year FU	*p*
	(*n* = 23 029)	(*n* = 1604)	(n = 14 228)	
Age (years)	67.7 ± 9.9	70.9 ± 9.4	66.8 ± 10.0	< 0.0001
Sex (male)	18 156 (78.8)	1352 (84.3)	11 127 (78.2)	< 0.0001
Hypertension	13 449 (58.4)	1075 (67.0)	7775 (54.6)	< 0.0001
Diabetes mellitus	7628 (33.1)	682 (42.5)	4341 (30.5)	< 0.0001
Heart failure	18 761 (81.5)	1469 (91.6)	11 179 (78.6)	< 0.0001
History of pulmonary edema	1572 (6.8)	237 (14.8)	743 (5.2)	< 0.0001
Dilated cardiomyopathy	15 983 (69.4)	1068 (66.6)	9861 (69.3)	0.03
Coronary artery disease	13 720 (59.6)	1172 (73.1)	8012 (56.3)	< 0.0001
Previous MI	2155 (9.4)	220 (13.7)	1062 (7.5)	< 0.0001
Previous PCI	5912 (25.7)	559 (34.9)	3237 (22.8)	< 0.0001
Previous CABG	2683 (11.7)	298 (18.6)	1501 (10.5)	< 0.0001
Aortic stenosis	1079 (4.7)	148 (9.2)	508 (3.6)	< 0.0001
Aortic regurgitation	916 (4.0)	112 (7.0)	486 (3.4)	< 0.0001
Mitral regurgitation	3896 (16.9)	400 (24.9)	2117 (14.9)	< 0.0001
Atrial fibrillation	9214 (40.0)	942 (58.7)	5143 (36.1)	< 0.0001
Left BBB	8510 (37.0)	545 (34.0)	5079 (35.7)	0.17
Right BBB	992 (4.3)	112 (7.0)	489 (3.4)	< 0.0001
Previous VF/sustained VT/cardiac arrest	0 (0.0)	0 (0.0)	0 (0.0)	—
Vascular disease	7567 (32.9)	759 (47.3)	4213 (29.6)	< 0.0001
Ischemic stroke	777 (3.4)	72 (4.5)	409 (2.9)	0.0004
Intracranial bleeding	181 (0.8)	26 (1.6)	91 (0.6)	< 0.0001
Smoker	3865 (16.8)	308 (19.2)	2183 (15.3)	0.0001
Dyslipidaemia	8701 (37.8)	693 (43.2)	5069 (35.6)	< 0.0001
Obesity	5129 (22.3)	384 (23.9)	2860 (20.1)	0.0003
Alcohol related diagnoses	1782 (7.7)	158 (9.9)	959 (6.7)	< 0.0001
Poor nutrition	898 (3.9)	146 (9.1)	339 (2.4)	< 0.0001
Chronic kidney disease	2201 (9.6)	341 (21.3)	976 (6.9)	< 0.0001
Lung disease	4117 (17.9)	417 (26.0)	2208 (15.5)	< 0.0001
Sleep apnoea syndrome	2377 (10.3)	193 (12.0)	1282 (9.0)	0.0001
Liver disease	1027 (4.5)	150 (9.4)	452 (3.2)	< 0.0001
Thyroid diseases	2125 (9.2)	226 (14.1)	1120 (7.9)	< 0.0001
Inflammatory disease	1317 (5.7)	154 (9.6)	636 (4.5)	< 0.0001
Anaemia	2181 (9.5)	331 (20.6)	973 (6.8)	< 0.0001
Previous cancer	1993 (8.7)	210 (13.1)	965 (6.8)	< 0.0001
Cognitive impairment	219 (1.0)	33 (2.1)	104 (0.7)	< 0.0001

*Note:* Values are *n* (%) or mean ± SD.

Abbreviations: CABG, coronary artery bypass graft; COPD, chronic obstructive pulmonary disease; ICD, implantable cardioverter defibrillator; LBBB, left bundle branch block; PCI, percutaneous coronary intervention; RBBB, right bundle branch block; SD, standard deviation.

**Figure 2:**
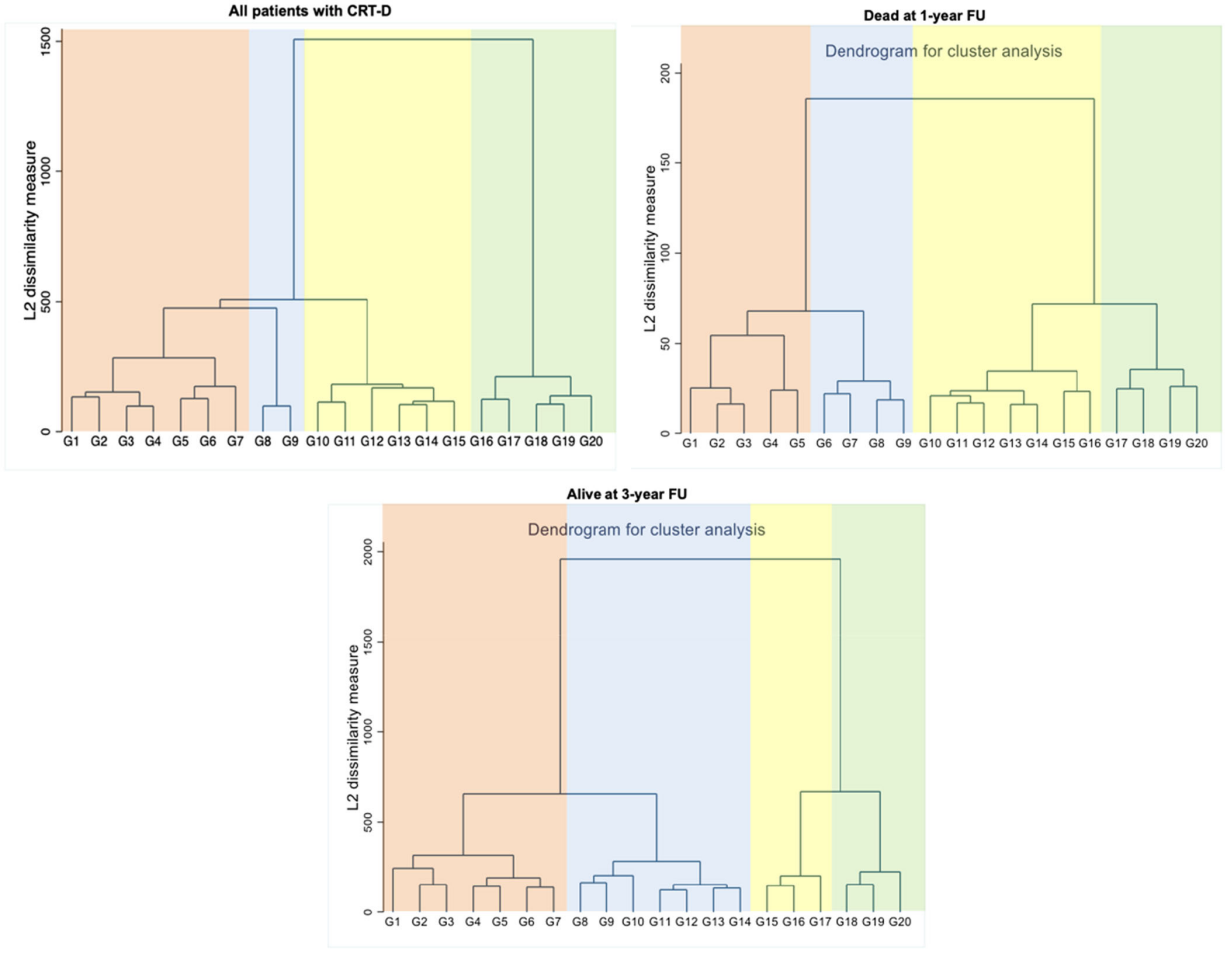
Dendrogram generated by hierarchical clustering process showing the four clusters in the patients from the 50% random sample (*n* = 11 514) of those with CRT‐D, in patients with CRT‐D dead at 1‐year FU, in patients with CRT‐D alive at 3‐year FU. The dendrogram graph is the visual representation of the hierarchical clustering process. Vertical lines are clusters that are joined together, and the position of the line on the scale indicates the distance at which the clusters were joined. Variables missing greater than 25% of data were excluded.

**Figure 3:**
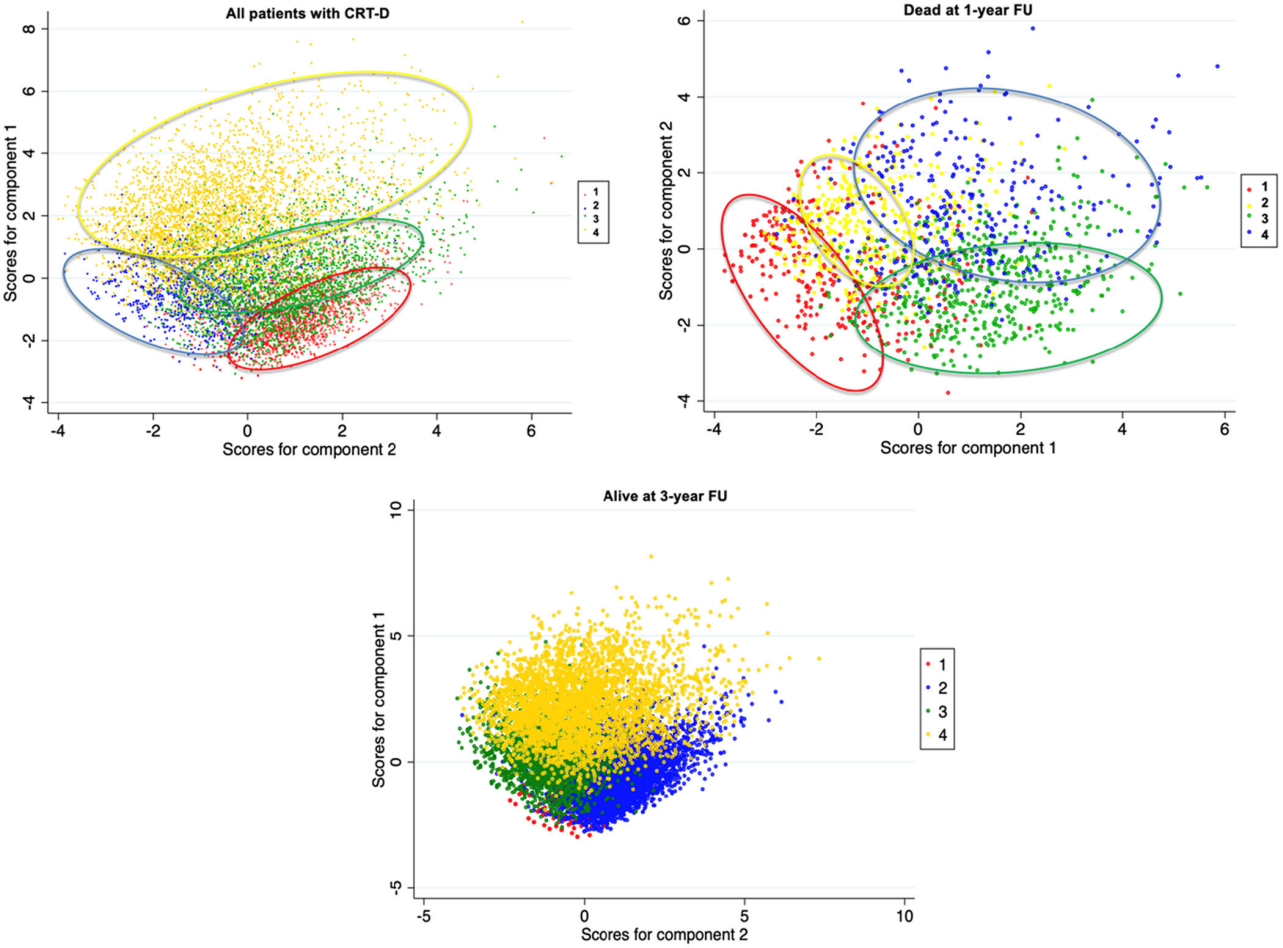
2D projection of the classification according to the four clusters in the patients from the 50% random sample (*n* = 11 514) of those with CRT‐D, in patients with CRT‐D dead at 1‐year FU, in patients with CRT‐D alive at 3‐year FU after reduction of dimension using principal component analysis.

To detect phenotypic categories of patients with CRT‐D without prior knowledge of the outcome, we used the hierarchical clustering method (using Ward's linkage criterion). The algorithm starts with each element (i.e., patient) as a separate cluster, and then goes on with a “bottom‐up” approach, grouping each cluster with the most similar one, until all clusters become one. A dendrogram for each of the three cluster analyses was obtained to detect the distance at each interaction of the clustering process (Figure 2). A small distance means that the clusters identified are similar and large values of the distance suggest that the two combined clusters are heterogenous. We did not prespecify the determination of the number of clusters. When analyzing the dendrograms, the groupings were more discordant after being expanded to four clusters. Clusters were derived independently within each patient group (groups 1, 2, and 3) using unsupervised hierarchical clustering. Therefore, the numeric labels assigned to clusters (cluster 1, 2, 3, 4) do not imply direct statistical equivalence across groups. However, the interpretation of cluster correspondence was based on the similarity of clinical phenotypes, which were found to be consistent across the three groups. Visual inspection of 2D projections and comparison of baseline characteristics supported this phenotypic alignment (Figure 3). Finally, we evaluated the association between identified clusters, baseline features, and clinical outcomes.

### 2.4 Statistical Analysis

Continuous variables are reported using mean ± standard deviation or median (interquartile range), while qualitative variables are reported as counts and percentages. The distribution of continuous variables was assessed using the Kolmogorov–Smirnov test. Parametric or non‐parametric tests were used to make comparisons: the Wilcoxon signed‐rank and Kruskal–Wallis tests for comparison of continuous values between two independent groups, and the *χ* ^2^ test for categorical data. The Kaplan–Meier curves considered patients from enrolment to the last follow‐up or death. We performed unadjusted and multivariable‐adjusted Cox analyses to evaluate the association between clusters and all‐cause death at 1 year and during the whole follow‐up. Results are reported as hazard ratios (HRs) and 95% confidence intervals (CIs). Associations between clusters and death at 1 year and death during the whole follow‐up (FU) were evaluated using Cox regression analyses. Analyses were performed using STATA, version 16.0 (StataCorp, College Station, TX, USA). Statistical significance levels were two‐sided, and significant differences were expressed as *p* < 0.05. When multiple comparisons were performed, Bonferroni's adjustment of α error was applied.

### 2.5 Ethics Approval

A dedicated approval by an ethical committee was not needed since this analysis used a French reference methodology, MR‐004, for research not directly involving human persons, including studies and evaluations in the field of health and healthcare. The reference methodology MR‐004 provides a framework for the processing of personal data for the purposes of studies, evaluation, or research that do not meet the definition of research involving the human person, in particular studies relating to the re‐use of data. The research must be in the public interest. The data controller undertakes to collect only data that is strictly necessary and relevant to the objectives of the research. The informed consent of patients was considered unnecessary, as this was a retrospective analysis, and there was no impact on their care. Patients and the public were not involved in the design, conduct, reporting, or dissemination of this study.

## 3 Results

Among 36 015 HF patients with a CRT and at least 1 year of follow‐up seen in French hospitals between 2010 and 2019, 12 986 (36%) had a CRT‐P and 23 029 (63%) carried a CRT‐D. CRT‐P patients were excluded to ensure a more homogeneous study population focused on CRT‐D implanted for primary prevention.

CRT‐D recipients were divided in three groups. Group 1 included 11 514 patients and was a 50% random sample of all patients with CRT‐D. Group 2 included 1604 patients who were dead after the first year of follow‐up. Group 3 comprised 14 228 patients who were alive at 3 years of follow‐up. We reported in Table 1 baseline clinical characteristics according to the three groups (total cohort, dead at 1‐year follow‐up, alive at 3 years follow‐up).

We conducted three different cluster analyses on the three groups identified. The analyses found four patient clusters for each group, as shown in the dendrograms (Figure 2). Figure 3 shows the projection of the classification, according to the four phenotypes identified after reduction of dimension using principal component analysis. Cluster 4 can be clearly distinguished from the other clusters. Despite areas of overlap, the four clusters maintain their homogeneity both in the 50% random sample of CRT‐D patients (group 1) and in those patients dead at 1 year of follow‐up (group 2) (Figure 3). For each of the three groups formed, baseline clinical characteristics according to the four clusters identified were analyzed and are reported in Tables 2, 3, 4 for groups 1, 2, and 3 respectively.

**Table 2:** Characteristics of patients with CRT‐D according to patient clusters (cluster analysis #1 with 4 clusters).

	Cluster 1	Cluster 2	Cluster 3	Cluster 4	*p*	Total
	(*n* = 3262)	(*n* = 1361)	(*n* = 3093)	(*n* = 3798)		(*n* = 11 514)
Age (years)	65.6 ± 11.1	67.5 ± 9.8	67.5 ± 9.3	70.2 ± 8.7	< 0.0001	67.8 ± 9.9
Sex (male)	2079 (63.7)	1290 (94.8)	2343 (75.8)	3386 (89.2)	< 0.0001	9098 (79.0)
Hypertension	1090 (33.4)	214 (15.7)	2348 (75.9)	3095 (81.5)	< 0.0001	6747 (58.6)
Diabetes mellitus	334 (10.2)	186 (13.7)	1375 (44.5)	1907 (50.2)	< 0.0001	3802 (33.0)
Heart failure	2652 (81.3)	942 (69.2)	2414 (78.0)	3385 (89.1)	< 0.0001	9393 (81.6)
History of pulmonary edema	170 (5.2)	39 (2.9)	161 (5.2)	437 (11.5)	< 0.0001	807 (7.0)
Dilated cardiomyopathy	2966 (90.9)	406 (29.8)	2656 (85.9)	1962 (51.7)	< 0.0001	7990 (69.4)
Coronary artery disease	617 (18.9)	1149 (84.4)	1459 (47.2)	3679 (96.9)	< 0.0001	6904 (60.0)
Previous MI	13 (0.4)	70 (5.1)	51 (1.6)	969 (25.5)	< 0.0001	1103 (9.6)
Previous PCI	76 (2.3)	249 (18.3)	335 (10.8)	2320 (61.1)	< 0.0001	2980 (25.9)
Previous CABG	15 (0.5)	276 (20.3)	70 (2.3)	1008 (26.5)	< 0.0001	1369 (11.9)
Aortic stenosis	71 (2.2)	40 (2.9)	143 (4.6)	298 (7.8)	< 0.0001	552 (4.8)
Aortic regurgitation	127 (3.9)	33 (2.4)	81 (2.6)	239 (6.3)	< 0.0001	480 (4.2)
Mitral regurgitation	601 (18.4)	107 (7.9)	382 (12.4)	887 (23.4)	< 0.0001	1977 (17.2)
Atrial fibrillation	1075 (33.0)	491 (36.1)	1204 (38.9)	1882 (49.6)	< 0.0001	4652 (40.4)
Left BBB	1129 (34.6)	287 (21.1)	1424 (46.0)	1435 (37.8)	< 0.0001	4275 (37.1)
Right BBB	80 (2.5)	21 (1.5)	110 (3.6)	291 (7.7)	< 0.0001	502 (4.4)
Previous VF/sustained VT/cardiac arrest	0 (0.0)	0 (0.0)	0 (0.0)	0 (0.0)	1	0 (0.0)
Vascular disease	123 (3.8)	425 (31.2)	397 (12.8)	2861 (75.3)	< 0.0001	3806 (33.1)
Ischemic stroke	82 (2.5)	18 (1.3)	123 (4.0)	164 (4.3)	< 0.0001	387 (3.4)
Intracranial bleeding	17 (0.5)	4 (0.3)	33 (1.1)	30 (0.8)	0.01	84 (0.7)
Smoker	344 (10.5)	85 (6.2)	573 (18.5)	968 (25.5)	< 0.0001	1970 (17.1)
Dyslipidaemia	223 (6.8)	122 (9.0)	1511 (48.9)	2439 (64.2)	< 0.0001	4295 (37.3)
Obesity	184 (5.6)	114 (8.4)	994 (32.1)	1216 (32.0)	< 0.0001	2508 (21.8)
Alcohol related diagnoses	254 (7.8)	34 (2.5)	306 (9.9)	279 (7.3)	< 0.0001	873 (7.6)
Poor nutrition	114 (3.5)	17 (1.2)	119 (3.8)	221 (5.8)	< 0.0001	471 (4.1)
Chronic kidney disease	214 (6.6)	48 (3.5)	205 (6.6)	641 (16.9)	< 0.0001	1108 (9.6)
Lung disease	396 (12.1)	166 (12.2)	575 (18.6)	930 (24.5)	< 0.0001	2067 (18.0)
Sleep apnoea syndrome	185 (5.7)	49 (3.6)	381 (12.3)	574 (15.1)	< 0.0001	1189 (10.3)
Liver disease	118 (3.6)	18 (1.3)	162 (5.2)	217 (5.7)	< 0.0001	515 (4.5)
Thyroid diseases	315 (9.7)	57 (4.2)	264 (8.5)	411 (10.8)	< 0.0001	1047 (9.1)
Inflammatory disease	137 (4.2)	21 (1.5)	230 (7.4)	295 (7.8)	< 0.0001	683 (5.9)
Anaemia	206 (6.3)	38 (2.8)	208 (6.7)	648 (17.1)	< 0.0001	1100 (9.6)
Previous cancer	237 (7.3)	46 (3.4)	284 (9.2)	423 (11.1)	< 0.0001	990 (8.6)
Cognitive impairment	14 (0.4)	7 (0.5)	41 (1.3)	51 (1.3)	< 0.0001	113 (1.0)

*Note:* Values are *n* (%) or mean ± SD.

Abbreviations: CABG, coronary artery bypass graft; COPD, chronic obstructive pulmonary disease; ICD, implantable cardioverter defibrillator; LBBB, left bundle branch block; PCI, percutaneous coronary intervention; RBBB, right bundle branch block; SD, standard deviation.

**Table 3:** Baseline characteristics of patients with CRT‐D dead at 1‐year FU seen in French hospitals (2010–2019) according to patient clusters (cluster analysis #2).

	Cluster 1	Cluster 2	Cluster 3	Cluster 4	*p*	Total
	(*n* = 375)	(*n* = 387)	(*n* = 517)	(*n* = 325)		(*n* = 1604)
Age (years)	67.7 ± 11.3	73.7 ± 8.2	73.6 ± 7.8	66.9 ± 8.1	< 0.0001	70.9 ± 9.4
Sex (male)	305 (81.3)	314 (81.1)	458 (88.6)	275 (84.6)	0.002	1352 (84.3)
Hypertension	71 (18.9)	289 (74.7)	443 (85.7)	272 (83.7)	< 0.0001	1075 (67.0)
Diabetes mellitus	68 (18.1)	154 (39.8)	243 (47.0)	217 (66.8)	< 0.0001	682 (42.5)
Heart failure	299 (79.7)	371 (95.9)	486 (94.0)	313 (96.3)	< 0.0001	1469 (91.6)
History of pulmonary edema	61 (16.3)	34 (8.8)	80 (15.5)	62 (19.1)	0.0002	237 (14.8)
Dilated cardiomyopathy	208 (55.5)	351 (90.7)	219 (42.4)	290 (89.2)	< 0.0001	1068 (66.6)
Coronary artery disease	212 (56.5)	177 (45.7)	514 (99.4)	269 (82.8)	< 0.0001	1172 (73.1)
Previous MI	42 (11.2)	5 (1.3)	145 (28.0)	28 (8.6)	< 0.0001	220 (13.7)
Previous PCI	48 (12.8)	26 (6.7)	365 (70.6)	120 (36.9)	< 0.0001	559 (34.9)
Previous CABG	34 (9.1)	16 (4.1)	215 (41.6)	33 (10.2)	< 0.0001	298 (18.6)
Aortic stenosis	10 (2.7)	44 (11.4)	62 (12.0)	32 (9.8)	< 0.0001	148 (9.2)
Aortic regurgitation	9 (2.4)	41 (10.6)	51 (9.9)	11 (3.4)	< 0.0001	112 (7.0)
Mitral regurgitation	55 (14.7)	115 (29.7)	172 (33.3)	58 (17.8)	< 0.0001	400 (24.9)
Atrial fibrillation	153 (40.8)	284 (73.4)	328 (63.4)	177 (54.5)	< 0.0001	942 (58.7)
Left BBB	79 (21.1)	172 (44.4)	175 (33.8)	119 (36.6)	< 0.0001	545 (34.0)
Right BBB	16 (4.3)	21 (5.4)	52 (10.1)	23 (7.1)	0.001	112 (7.0)
Previous VF/sustained VT/cardiac arrest	0 (0.0)	0 (0.0)	0 (0.0)	0 (0.0)	1	0 (0.0)
Vascular disease	106 (28.3)	83 (21.4)	405 (78.3)	165 (50.8)	< 0.0001	759 (47.3)
Ischemic stroke	4 (1.1)	18 (4.7)	28 (5.4)	22 (6.8)	0.0005	72 (4.5)
Intracranial bleeding	0 (0.0)	8 (2.1)	5 (1.0)	13 (4.0)	0.0001	26 (1.6)
Smoker	23 (6.1)	36 (9.3)	119 (23.0)	130 (40.0)	< 0.0001	308 (19.2)
Dyslipidaemia	32 (8.5)	122 (31.5)	346 (66.9)	193 (59.4)	< 0.0001	693 (43.2)
Obesity	24 (6.4)	69 (17.8)	119 (23.0)	172 (52.9)	< 0.0001	384 (23.9)
Alcohol related diagnoses	26 (6.9)	20 (5.2)	43 (8.3)	69 (21.2)	< 0.0001	158 (9.9)
Poor nutrition	20 (5.3)	41 (10.6)	63 (12.2)	22 (6.8)	0.0004	146 (9.1)
Chronic kidney disease	21 (5.6)	79 (20.4)	150 (29.0)	91 (28.0)	< 0.0001	341 (21.3)
Lung disease	41 (10.9)	54 (14.0)	144 (27.9)	178 (54.8)	< 0.0001	417 (26.0)
Sleep apnoea syndrome	18 (4.8)	26 (6.7)	40 (7.7)	109 (33.5)	< 0.0001	193 (12.0)
Liver disease	32 (8.5)	30 (7.8)	29 (5.6)	59 (18.2)	< 0.0001	150 (9.4)
Thyroid diseases	35 (9.3)	50 (12.9)	94 (18.2)	47 (14.5)	0.001	226 (14.1)
Inflammatory disease	14 (3.7)	51 (13.2)	41 (7.9)	48 (14.8)	< 0.0001	154 (9.6)
Anaemia	28 (7.5)	44 (11.4)	165 (31.9)	94 (28.9)	< 0.0001	331 (20.6)
Previous cancer	32 (8.5)	50 (12.9)	79 (15.3)	49 (15.1)	0.01	210 (13.1)
Cognitive impairment	2 (0.5)	7 (1.8)	17 (3.3)	7 (2.2)	0.02	33 (2.1)

*Note:* Values are *n* (%) or mean ± SD.

Abbreviations: CABG, coronary artery bypass graft; COPD, chronic obstructive pulmonary disease; ICD, implantable cardioverter defibrillator; LBBB, left bundle branch block; PCI, percutaneous coronary intervention; RBBB, right bundle branch block; SD, standard deviation

**Table 4:** Baseline characteristics of patients with CRT‐D alive at 3‐year FU seen in French hospitals (2010–2019) according to patient clusters (cluster analysis #3).

	Cluster 1	Cluster 2	Cluster 3	Cluster 4	*p*	Total
	(*n* = 1855)	(*n* = 5551)	(*n* = 2894)	(*n* = 3928)		(*n* = 14 228)
Age (years)	64.2 ± 11.4	65.3 ± 10.2	68.1 ± 9.2	68.9 ± 8.7	< 0.0001	66.8 ± 10.0
Sex (male)	1434 (77.3)	3773 (68.0)	2550 (88.1)	3370 (85.8)	< 0.0001	11127 (78.2)
Hypertension	131 (7.1)	3210 (57.8)	1044 (36.1)	3390 (86.3)	< 0.0001	7775 (54.6)
Diabetes mellitus	69 (3.7)	1526 (27.5)	860 (29.7)	1886 (48.0)	< 0.0001	4341 (30.5)
Heart failure	1023 (55.1)	4511 (81.3)	2091 (72.3)	3554 (90.5)	< 0.0001	11179 (78.6)
History of pulmonary edema	0 (0.0)	269 (4.8)	56 (1.9)	418 (10.6)	< 0.0001	743 (5.2)
Dilated cardiomyopathy	1279 (68.9)	5110 (92.1)	1319 (45.6)	2153 (54.8)	< 0.0001	9861 (69.3)
Coronary artery disease	191 (10.3)	1327 (23.9)	2711 (93.7)	3783 (96.3)	< 0.0001	8012 (56.3)
Previous MI	0 (0.0)	24 (0.4)	139 (4.8)	899 (22.9)	< 0.0001	1062 (7.5)
Previous PCI	0 (0.0)	148 (2.7)	959 (33.1)	2130 (54.2)	< 0.0001	3237 (22.8)
Previous CABG	0 (0.0)	57 (1.0)	555 (19.2)	889 (22.6)	< 0.0001	1501 (10.5)
Aortic stenosis	1 (0.1)	199 (3.6)	60 (2.1)	248 (6.3)	< 0.0001	508 (3.6)
Aortic regurgitation	0 (0.0)	231 (4.2)	32 (1.1)	223 (5.7)	< 0.0001	486 (3.4)
Mitral regurgitation	4 (0.2)	1066 (19.2)	182 (6.3)	865 (22.0)	< 0.0001	2117 (14.9)
Atrial fibrillation	360 (19.4)	2074 (37.4)	800 (27.6)	1909 (48.6)	< 0.0001	5143 (36.1)
Left BBB	385 (20.8)	2277 (41.0)	914 (31.6)	1503 (38.3)	< 0.0001	5079 (35.7)
Right BBB	4 (0.2)	146 (2.6)	81 (2.8)	258 (6.6)	< 0.0001	489 (3.4)
Previous VF/sustained VT/cardiac arrest	0 (0.0)	0 (0.0)	0 (0.0)	0 (0.0)	1	0 (0.0)
Vascular disease	6 (0.3)	340 (6.1)	1073 (37.1)	2794 (71.1)	< 0.0001	4213 (29.6)
Ischemic stroke	2 (0.1)	210 (3.8)	24 (0.8)	173 (4.4)	< 0.0001	409 (2.9)
Intracranial bleeding	0 (0.0)	48 (0.9)	11 (0.4)	32 (0.8)	< 0.0001	91 (0.6)
Smoker	28 (1.5)	823 (14.8)	262 (9.1)	1070 (27.2)	< 0.0001	2183 (15.3)
Dyslipidaemia	14 (0.8)	1511 (27.2)	723 (25.0)	2821 (71.8)	< 0.0001	5069 (35.6)
Obesity	15 (0.8)	1207 (21.7)	241 (8.3)	1397 (35.6)	< 0.0001	2860 (20.1)
Alcohol related diagnoses	2 (0.1)	551 (9.9)	83 (2.9)	323 (8.2)	< 0.0001	959 (6.7)
Poor nutrition	1 (0.1)	138 (2.5)	28 (1.0)	172 (4.4)	< 0.0001	339 (2.4)
Chronic kidney disease	11 (0.6)	335 (6.0)	73 (2.5)	557 (14.2)	< 0.0001	976 (6.9)
Lung disease	46 (2.5)	937 (16.9)	188 (6.5)	1037 (26.4)	< 0.0001	2208 (15.5)
Sleep apnoea syndrome	9 (0.5)	473 (8.5)	94 (3.2)	706 (18.0)	< 0.0001	1282 (9.0)
Liver disease	1 (0.1)	195 (3.5)	41 (1.4)	215 (5.5)	< 0.0001	452 (3.2)
Thyroid diseases	22 (1.2)	556 (10.0)	95 (3.3)	447 (11.4)	< 0.0001	1120 (7.9)
Inflammatory disease	11 (0.6)	286 (5.2)	56 (1.9)	283 (7.2)	< 0.0001	636 (4.5)
Anaemia	5 (0.3)	343 (6.2)	53 (1.8)	572 (14.6)	< 0.0001	973 (6.8)
Previous cancer	7 (0.4)	454 (8.2)	105 (3.6)	399 (10.2)	< 0.0001	965 (6.8)
Cognitive impairment	2 (0.1)	36 (0.6)	7 (0.2)	59 (1.5)	< 0.0001	104 (0.7)

*Note:* Values are *n* (%) or mean ± SD.

Abbreviations: CABG, coronary artery bypass graft; COPD, chronic obstructive pulmonary disease; ICD, implantable cardioverter defibrillator; LBBB, left bundle branch block; PCI, percutaneous coronary intervention; RBBB, right bundle branch block; SD, standard deviation.

**Figure 4:**
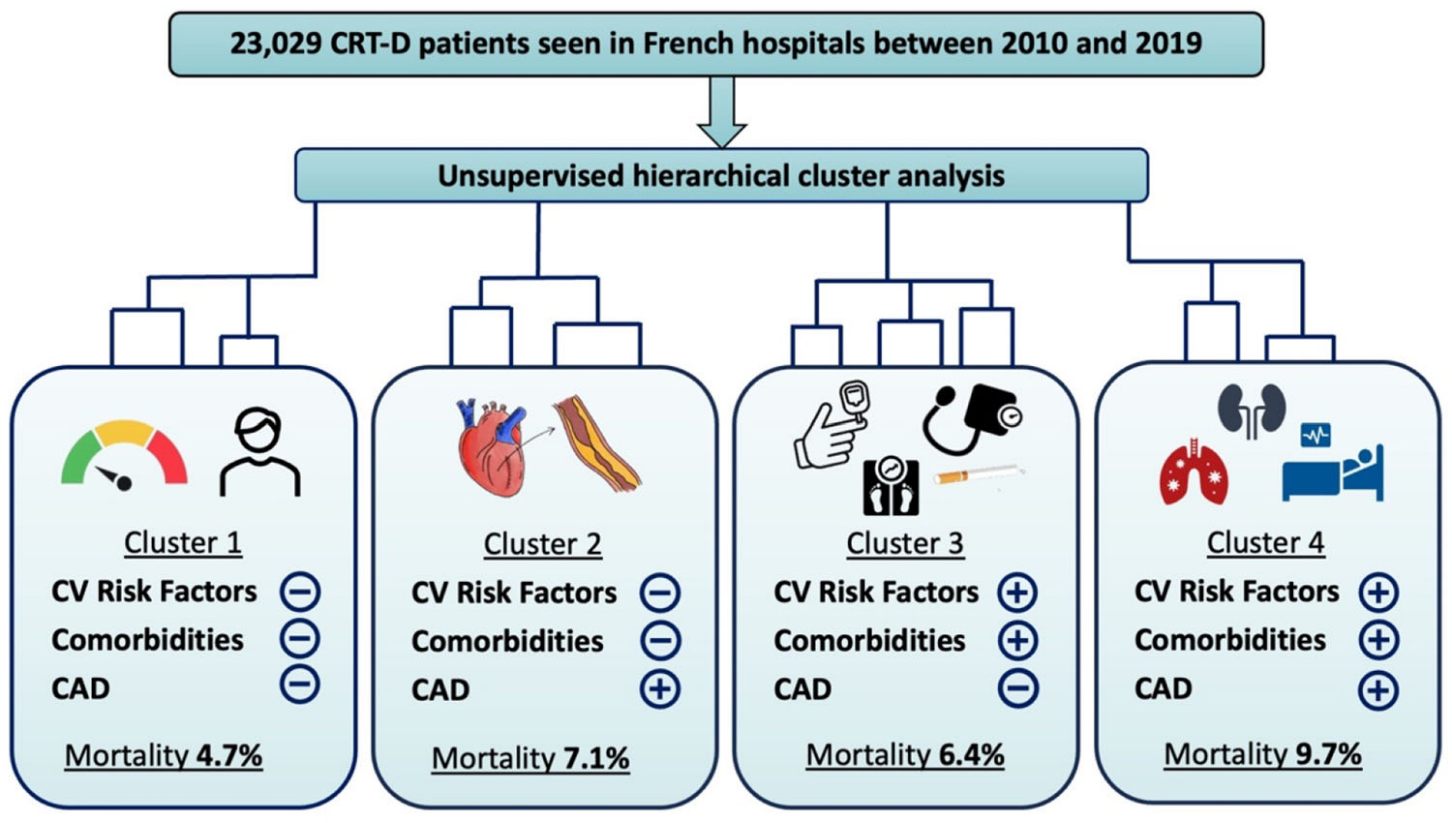
Graphical abstract showing the 4 clusters identified in CRT‐D patients.

To facilitate interpretation, the four clusters can be broadly described as follows: (1) low CAD, risk factors and comorbidities, (2) CAD with low risk factors and comorbidities, (3) risk factors and comorbidities with low CAD phenotype, and (4) high CAD, risk factors and comorbidities phenotype (Figure 4).

### 3.1 Clusters

#### 3.1.1 Cluster 1 (Low CAD, Risk Factors, and Comorbidities)

In groups 1, 2, and 3, cluster 1 included 3262 (28%), 375 (23%), and 1855 (13%) patients, respectively. According to the clinical features reported, compared to other clusters, patients were younger (mean age 65.6 ± 11.1 years in group 1, 67.7 ± 11.3 in group 2 and 64.2 ± 11.4 in group 3) with a higher proportion of female patients, low prevalence of hypertension (33.3% in group 1, 18.9% in group 2, and 7.1% in group 3), diabetes (10.2% in group 1, 18.1% in group 2, and 3.7% in group 3), dyslipidemia (6.8% in group 1, 8.5% in group 2, and 0.8% in group 3), and obesity (5.6% in group 1, 6.4% in group 2, and 0.8% in group 3). There was a lower prevalence of ischemic cardiomyopathy/coronary artery disease (CAD) (18.9% in group 1, 56.5% in group 2, and 10.3% in group 3) and atrial fibrillation (AF) (33% in group 1, 40.8% in group 2, and 19.4% in group 3). Moreover, these patients had a lower burden of comorbidities, specifically CKD (6.6% in group 1, 5.6% in group 2, and 0.6% in group 3), lung disease (12.1% in group 1, 10.9% in group 2, and 2.5% in group 3) and liver disease (3.6% in group 1, 8.5% in group 2, and 0.1% in group 3) (*p* < 0.0001 vs. clusters 2, 3, and 4).

#### 3.1.2 Cluster 2 (CAD With Low Risk Factors and Comorbidities)

In groups 1, 2, and 3, cluster 2 was composed of 1361 (12%), 387 (24%), and 5551 (39%) patients, respectively. In this cluster, CRT‐D recipients tended to have a male predominance, mainly in group 1 (94.8%). Compared to the other clusters, they had a low prevalence of cardiovascular (CV) risk factors (hypertension: 15.7%, diabetes: 13.7% and dyslipidemia: 9% in group 1) and comorbidities (CKD: 3.5%, lung disease: 12.2%, and liver disease: 1.3% in group 1). However, the main clinical feature was the high prevalence of CAD (84.4% in group 1), associated with a high proportion of patients who underwent coronary artery bypass graft (CABG) (20.3%). These features were mainly observed in group 1 (*p* < 0.0001 vs. clusters 2, 3, and 4), while in the other two groups this clinical phenotype was mitigated and less evident.

#### 3.1.3 Cluster 3 (Risk Factors and Comorbidities With Low CAD)

Cluster 3 included 3093 in group 1 (27%), 517 in group 2 (32%), and 2894 in group 3 (20%). The clinical phenotype was characterized by a high prevalence of hypertension (75.9% in group 1, 85.7% in group 2, but 36.1% in group 3), diabetes mellitus (44.5% in group 1, 47% in group 2, 29.7% in group 3), dyslipidemia (48.9% in group 1, 66.9% in group 2, 25% in group 3). Patients in this cluster had a high prevalence of CAD particularly in group 3 (47.2% in group 1, 99.4% in group 2, and 93.7% in group 3), however, compared to cluster 2, there was a higher proportion of patients who underwent percutaneous coronary intervention (PCI) (10.8% in group 1, 70.6% in group 2, and 33.1% in group 3). The comorbidity burden was low, but only compared to cluster 4 and mainly in group 1 and group 3. CKD was reported in 6.6%, 29%, 2.5% in groups 1, 2, and 3, respectively, lung disease in 18.6%, 27.9%, and 6.5% in groups 1, 2, and 3, respectively and liver disease in 5.2%, 5.6%, and 1.4% in group 1, 2, and 3 respectively. Vascular disease was common (12.8% in group 1, 78.3% in group 2, and 37.1% in group 3).

#### 3.1.4 Cluster 4 (High CAD, Risk Factors, and Comorbidities)

This cluster was composed of 3798 in group 1 (33%), 325 in group 2 (20%), and 3928 in group 3 (28%). Mean age was 70.2 ± 8.7 years in group 1, 66.9 ± 8.1 in group 2, and 68.9 ± 8.7 in group 3 (identifying an older population compared to other clusters, *p* < 0.0001), a male majority (89.2%, 84.6%, 85.8% in group 1,2, and 3, respectively). There was a high burden of CV risk factors (hypertension (81.5, 83.7, 86.3), diabetes (50.2, 66.8, 48), dyslipidemia (64.2, 59.4, 71.8) and obesity (32, 52.9, 35.6), together with a high burden of comorbidity (CKD 16.9, 28.0, 14.2, lung disease 24.5, 54.8, 26.4, liver disease 5.7, 18.2, 5.5, cancer 11.1, 15.1, 10.2 and vascular disease 75.3, 50.8, 71.1). In all three groups, the proportion of CAD was relevant (96.9, 82.8, 96.3), and a significant proportion of patients was affected by AF (49.6, 54.5, 48.6). Like cluster 1, this complex phenotype was consistent in all three groups considered (*p* < 0.0001 vs. clusters 2, 3, and 4). Of note, in cluster 4, a high burden of both CAD and dilated cardiomyopathy was observed. This reflects the real‐world overlap between ischemic and nonischemic cardiomyopathy definitions, where patients with diffuse ischemic damage may present a dilated phenotype or may be labeled as DCM despite the presence of significant CAD.

### 3.2 Associations With Clinical Outcomes

Clusters 2, 3, and 4, compared to cluster 1, were independently associated with outcomes considered in our analysis. Table 5 reports the crude rates and annual risk of all‐cause death between clusters. Over a median follow‐up of 3.9 years (IQR 2.1–6.1) years, all‐cause death at 1‐year follow‐up occurred more commonly in cluster 4 (9.7%), cluster 2 (7.1%), and cluster 3 (6.4%) compared to cluster 1 (4.7%) (all *p* < 0.01). The same associations were observed when considering death during the whole follow‐up. Indeed, the percentage of deaths during whole follow‐up was 36.6% for cluster 4, 33.6% for cluster 2, 26.9% for cluster 3, all significantly higher compared to cluster 1 (25.5%) (all *p* < 0.01).

**Table 5:** The number of incident all‐cause deaths, risk time, and incidence rate according to cluster analysis in all patients treated with CRT‐D (cluster analysis #1).

	Number of patients	Person‐years	Death at 1‐year FU	Death during the whole FU	Incidence rate (%/year, 95% CI)	HR vs. cluster 1, 95%CI	*p*
Cluster 1	3262	14 823	152 (4.7)	832 (25.5)	5.61 (5.24–6.01)	—	—
Cluster 2	1361	6 569	97 (7.1)	457 (33.6)	6.96 (6.35–7.63)	1.211 (1.080–1.358)	0.001
Cluster 3	3093	13 047	197 (6.4)	831 (26.9)	6.37 (5.95–6.82)	1.147 (1.042–1.263)	0.005
Cluster 4	3798	14 090	367 (9.7)	1391 (36.6)	9.87 (9.37–10.41)	1.796 (1.648–1.957)	< 0.0001

When analyzing the hazard ratio (HR)s compared with Cluster 1, Clusters 2, 3, and 4 were independently associated with an increased risk of all‐cause death at 1‐year FU and during the whole FU (Cluster 2: hazard ratio (HR) 1.21, 95% confidence interval (CI) 1.08–1.36; Cluster 3: HR 1.15, 95% CI 1.04–1.26; and Cluster 4: HR 1.79, 95% CI 1.65–1.96).

**Figure 5:**
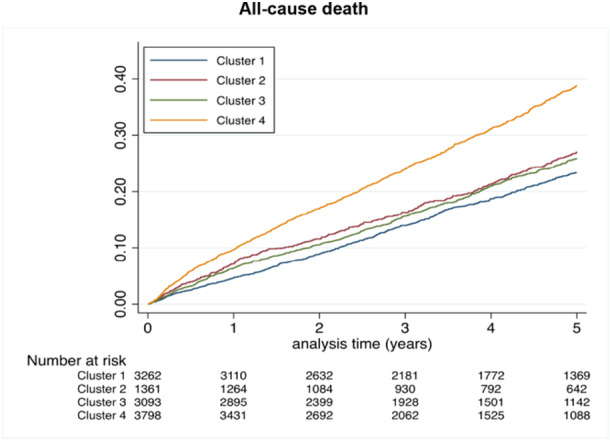
Incidence of all‐cause death in patients with CRT‐D stratified in the 4 clusters from cluster analysis #1.

Figure 5 shows the Kaplan–Meier curves, which represent the incidence of all‐cause death in patients with CRT‐D across the different four clusters from analysis #1. Cluster 1 had a significantly better prognosis compared to other clusters. The occurrence of the primary outcome was significantly higher in cluster 4, but also in cluster 3 and 2 when compared to cluster 1.

We tested the proportional hazards assumption for all variables included in the Cox regression models using Schoenfeld residuals. No significant violations of the proportional hazards assumption were observed (*p* value for global test 0.295, and *p* value ranging from 0.121 to 0.987 for each variable).

## 4 Discussion

In the present study, we provide clinical insights on the CRT‐D patient population. In particular, we found that: (1) patients who died after 1 year after CRT‐D implantation are significantly different compared to patients who were alive at 3 years of follow‐up, being older, having a higher prevalence of CV risk factors, CAD, AF, and comorbidity; (2) cluster analysis led to the identification of four statistically driven groups of CRT‐D patients with distinct clinical phenotypes; (3) the clusters were significantly different in terms of cardiovascular diseases, demographic characteristics, CV risk factors, and co‐morbidities; and (4) the clusters identified were independently associated with different risks for all‐cause death.

Depending on the definition of CRT response, about one‐third of CRT recipients are considered non‐responders [13]. Response to CRT might be essentially defined in terms of functional improvement (New York Heart Association (NYHA) functional class, 6 min walking test distance and quality of life) [14], echocardiographic remodeling [13], and clinical outcomes [4]. There is no consensus on how and when to evaluate such a response. Moreover, for primary prevention, there is an active debate on whether to implant a CRT‐D or a CRT‐P [15], since evidence is conflicting and the benefit from implantable cardioverter defibrillator (ICD), especially in the elderly, is controversial [16]. CRT‐D response cannot be considered as a dichotomous (yes/no) event, but rather as a continuum. For this reason, CRT‐D response prediction cannot rely solely on a single parameter. Previous studies, based on scores, tried to identify those patients likely to derive little benefit from CRT‐D. Specifically, the CRT‐D futility score [17] outperformed the Goldenberg and EAARN scores [18, 19] for identifying futility. Although promising and relatively easy to apply in clinical practice, these methods hold intrinsic limitations, mainly related to the fact that they can consider only a few parameters, while CRT‐D candidates are a heterogenous and complex population, which requires a multiparametric approach.

Cluster analysis has been developed to better understand heterogeneous groups of patients and to find clinical phenotypes associated with different outcomes [10, 20]. Few cluster analyses have been conducted in HF patients with CRT‐D. Gallard et al. [11] evaluated 250 patients before CRT implantation, and they identified five clusters based on echocardiographic strain features. Cluster 5 had the highest percentage of responders in terms of a decrease of ≥ 15% in left ventricular end‐systolic volume, and it was associated with the lowest event rate after implantation. This low‐risk cluster was characterized by high strain integrals compared to cluster 1, which had the worst responder rate and the highest event rate. The authors concluded that longitudinal strain curves could be promising in improving patient characterization and selection for CRT.

In our large French national registry, we sought to investigate whether an unsupervised cluster analysis can detect, among CRT‐D patients, clinical phenotypes associated with different risks of all‐cause death and whether these phenotypes are consistent in patients who died within 1 year of follow‐up and in patients alive after 3 years. Identifying clinical profiles could be crucial for a better selection of patients before implantation and to maximize the response to CRT‐D. We have excluded patients with a history of ventricular arrhythmia, or cardiac arrest to focus on a population receiving CRT‐D for primary prevention in heart failure. CRT‐P patients were excluded from the analysis to avoid confounding when comparing outcomes specifically related to CRT‐D therapy.

Cluster 1 identified patients with a low burden of CV risk factors and comorbidities and a low prevalence of CAD. This clinical profile was associated with the best response in terms of all‐cause death. The prevalence of patients from cluster 1, dead after the first year and during the whole follow‐up was significantly lower compared to other clusters, above all compared to cluster 4. Indeed, cluster 4 corresponds to a clinical phenotype considered at high risk of death and cardiovascular events because of the high prevalence of CAD, AF, and comorbidities. Nonetheless, the magnitude of effect for all‐cause mortality does not seem to be large, specifically it was statistically significant between cluster 1 and cluster 4, however the effect was blunted when considering the difference between cluster 2–3 and cluster 1.

Cardiovascular risk factors have been associated with a worse prognosis in patients with CRT‐D. Diabetes mellitus might cause metabolic, electrical, and endothelial dysfunction, contributing to heart failure progression and reduced CRT‐D response [21, 22]. The effect of hypertension following CRT‐D implantation has been a matter of debate. For example, Biton et al. [23] found that patients with low baseline systolic blood pressure (SBP) (defined as those with a median SBP of 100 mmHg) had a higher risk of mortality or HF with an ICD therapy alone compared to patients with a CRT‐D. The greater benefit from resynchronization therapy probably derives from the greater increase in SBP following implantation, while the benefit is attenuated when SBP is high at baseline. On the other hand, although hypertension has been associated with a reduced response to CRT, it has been demonstrated that prognosis in HF patients after CRT‐D implantation improves despite increasing CV risk factors burden over time [24].

The prevalence of CAD and ischemic cardiomyopathy, identified based on diagnostic codes recorded in medical records, was high in clusters 2 and 4. Specifically, mainly in cluster 4, there was a significant proportion of patients who had a myocardial infarction and underwent percutaneous coronary angiography (PCI). Traditionally, ischemic cardiomyopathy has been associated with less remodeling and echocardiographic response after CRT‐D implantation [25]. Rather than ischemic etiology itself, it seems that the scar burden plays a role in increasing the risk of death and in reducing the benefit from CRT‐D. Harb et al. [26] showed that a high scar percentage, identified by cardiac magnetic resonance, is associated with reduced left ventricular functional improvement and adverse events after resynchronization therapy, in both ischemic and nonischemic cardiomyopathy.

In cluster 4, about half of the patients had AF, both in those dead at 1 year of follow‐up and in those alive at 3 years. The prevalence of AF was significantly high (> 50% of patients) also in clusters 2 and 3, when considering those patients dead within the first year. AF may blunt CRT‐D response, reducing the percentage of biventricular pacing [27]. The presence of an irregular rhythm reduces the left ventricle capture and synchronization [28]. Moreover, since AF is a marker of HF severity, the lack of response following CRT‐D implantation might be related to the progression of the disease itself, independently of AF [29]. In these patients, atrio‐ventricular (AV) node ablation has been suggested as a potential strategy to improve CRT‐D response. In the APAF‐CRT trial [30] and in the APAF‐CRT mortality trial [31], AV junction ablation for patients with CRT and AF was superior to a rate‐control strategy in terms of HF hospitalization and all‐cause death.

In our analysis, comorbidities such as chronic kidney disease (CKD), considered as a binary variable (presence vs. absence), and lung disease, were significantly higher in cluster 4 and in those patients dead at 1 year. Moderate to severe renal impairment (CKD stages 3 to 5) has been independently associated with worst outcomes following CRT implantation [32], as well as chronic obstructive pulmonary disease (COPD) was found to be an independent predictor of nonresponse to resynchronization therapy [33]. The role of renal failure in this context is not completely clear. Leyva et al. [24] demonstrated that, over a 9‐year period, in a CRT patients' population, the prevalence of CKD increased significantly; however, total mortality kept decreasing over time after CRT implantation. Similarly, Zeitler et al. [34], analyzing the long‐term follow‐up of the MADIT‐CRT trial, found that the burden of comorbidity and CV risk factors did not compromise the benefit of CRT‐D compared to ICD alone. The mechanisms responsible for this are not completely clear, however these findings support the idea that the comorbidity burden should not be an absolute exclusion criterion for CRT‐D implantation.

In our analysis, we identified a group of patients (cluster 4), that compared to other clusters (1, 2, and 3) was associated with a significant increase in all‐cause death. Thus, our study highlights the need to better characterize the candidates to resynchronization therapy, not just to exclude them from implantation, but to manage risk factors and comorbidities, which might blunt the response to CRT‐D.

## 5 Limitations

This study was based on a registry and is subject to the limitations inherent in retrospective observational analyses. Moreover, clustering algorithm results are dependent on the underlying population, available clinical variables, and associated patterns of care in the community. Then, as cluster analysis depends on available data, the incorporation of more clinical characteristics, such as ejection fraction, QRS duration, brain natriuretic peptide might yield different results. However, since cluster analysis necessitates complete data on individual patients, we chose to drop variables with a greater than 25% missing data rate to ensure the quality of the data. Patients were included from 2010 to 2019, and treatment strategies and clinical practice have changed over time, partly limiting the generalization of the results to current clinical practice.

The study focuses on absolute all‐cause mortality, reporting the total number of deaths from any cause among the studied population; the study does not assess the relative benefit of CRT‐D. This means that, reporting overall mortality, it does not determine whether CRT specifically improves survival relative to other treatment options or a control group.

When considering comorbidities, a formal comorbidity index such as the Charlson could provide an additional quantitative metric; our approach integrated comorbidities directly into the unsupervised cluster analysis.

Although cluster analysis is not properly a predictive model, we aimed to demonstrate that this approach, which is an unsupervised method (outcome blinded), is able to distinguish relevant phenogroups regarding prognosis in cohort of patients implanted with CRT‐D devices for primary prevention. This complementary approach to usual clinical models may improve our understanding of clinical complexity and provide us new insights.

## 6 Conclusion

In this study on HF patients with CRT‐D, unsupervised cluster analysis identified four statistically driven groups of CRT recipients, with specific clinical phenotypes and associated with different risks for all‐cause death. Identifying those patients at higher risk of adverse events might help in the selection of patients with heart failure undergoing CRT‐D implantation, especially in relation to CRT nonresponse.

## Conflicts of Interest

G.Y.H.L. is a consultant and speaker for BMS/Pfizer, Boehringer Ingelheim, Daiichi‐Sankyo, Anthos. No fees are received personally. He is a National Institute for Health and Care Research (NIHR) Senior Investigator and co‐PI of the AFFIRMO project on multimorbidity in AF (Grant agreement No 899871), TARGET project on digital twins for personalised management of atrial fibrillation and stroke (grant agreement no 101136244) and ARISTOTELES project on artificial intelligence for management of chronic long term conditions (Grant agreement No 101080189), which are all funded by the EU's Horizon Europe Research and Innovation programme. AB has been a consultant or speaker for Astra‐Zeneca, Bayer, BMS/Pfizer, Medtronic, Vitorpharma, and Alnylam.

## Data Availability

The data that support the findings of this study are available from the corresponding author upon reasonable request.
